# Crosstalk within and beyond the Polycomb repressive system

**DOI:** 10.1083/jcb.202311021

**Published:** 2024-03-20

**Authors:** Tianyi Hideyuki Shi, Hiroki Sugishita, Yukiko Gotoh

**Affiliations:** 1 Graduate School of Pharmaceutical Sciences, The University of Tokyo, Tokyo, Japan; 2 International Research Center for Neurointelligence, The University of Tokyo, Tokyo, Japan

## Abstract

The development of multicellular organisms depends on spatiotemporally controlled differentiation of numerous cell types and their maintenance. To generate such diversity based on the invariant genetic information stored in DNA, epigenetic mechanisms, which are heritable changes in gene function that do not involve alterations to the underlying DNA sequence, are required to establish and maintain unique gene expression programs. Polycomb repressive complexes represent a paradigm of epigenetic regulation of developmentally regulated genes, and the roles of these complexes as well as the epigenetic marks they deposit, namely H3K27me3 and H2AK119ub, have been extensively studied. However, an emerging theme from recent studies is that not only the autonomous functions of the Polycomb repressive system, but also crosstalks of Polycomb with other epigenetic modifications, are important for gene regulation. In this review, we summarize how these crosstalk mechanisms have improved our understanding of Polycomb biology and how such knowledge could help with the design of cancer treatments that target the dysregulated epigenome.

## Teaser

Shi et al. review emerging concepts regarding crosstalk mechanisms of Polycomb repressive complexes and their implications in epigenetic therapies for cancer.

## Introduction

The development of multicellular organisms involves precisely regulated gene expression programs, the fidelity of which depends on not only transcription factors but also epigenetic mechanisms that involve mitotically heritable modifications of DNA, histones, and chromatin conformation. A highly conserved group of such epigenetic factors in multicellular organisms is formed by the Polycomb group (PcG) proteins. Originally discovered in *Drosophila* as a repression mechanism of ectopic HOX gene expression (Lewis, 1978), the Polycomb repressive system has evolved in vertebrates to play more diverse roles in development (Schuettengruber et al., 2017), and its dysfunction has been implicated in numerous human developmental disorders and cancer (Piunti and Shilatifard, 2021; Tamburri et al., 2022; Bölicke and Albert, 2022).

PcG proteins assemble into either Polycomb repressive complex 1 (PRC1) or PRC2. PRC1 has a heterodimeric core, consisting of the E3 ubiquitin ligase RING1B (or its less prominent paralog, RING1A), which catalyzes histone H2A lysine 119 monoubiquitylation (H2AK119ub), and one of the six PCGF proteins (PCGF1/2/3/4/5/6). The PRC2 core complex includes EZH2 (or EZH1), responsible for mono-, di-, and trimethylation of lysine 27 of histone H3 (H3K27me1/2/3), and the structural components EED, SUZ12, and RBBP4/7. Functional PRC1 and PRC2 complexes have their cores associated with additional accessory factors, which confer functional diversity on them (Gao et al., 2012; Kim and Kingston, 2022).

Notably, while early studies proposed a dual role of PRC1 in H2AK119ub deposition (de Napoles et al., 2004; Wang et al., 2004) and chromatin compaction (Francis et al., 2004; Grau et al., 2011), these activities were later found to be independent of each other (Eskeland et al., 2010) and were attributed to two disparate classes of PRC1, namely canonical PRC1 (cPRC1) and non-canonical PRC1 (ncPRC1), based on the mutually exclusive inclusion of CBX and RYBP subunits, respectively (Gao et al., 2012; Blackledge et al., 2014). cPRC1 contains PCGF2 or PCGF4 at the core and associates with one of the PHC proteins (PHC1/2/3) and one of the CBX proteins (CBX2/4/6/7/8), both of which contribute to cPRC1 nucleosome compaction and chromatin condensation activities (Isono et al., 2005, 2013; Lau et al., 2017; Plys et al., 2019; Jaensch et al., 2021). Relating to these activities, cPRC1 also mediates long-range chromatin interactions and the formation of PcG bodies, which are subnuclear compartments enriched for PcG proteins (Du et al., 2020; Boyle et al., 2020; Kundu et al., 2017; Bonev et al., 2017; Schoenfelder et al., 2015). PcG long-range interactions are partially dependent on the cyclin-dependent kinase module mediator complex (CKM–Mediator) (Dimitrova et al., 2022), but how these activities relate to gene regulation is not fully understood. cPRC1’s contribution to in vivo H2AK119 ubiquitylation is suggested to be very low given the limited catalytic activity of the RING1B–PCGF core complex, which is further inhibited by cPRC1’s accessory subunits, likely PHC1/2/3 (Blackledge et al., 2014; Gao et al., 2012). In contrast, ncPRC1, which has PCGF1/3/5/6 (or less commonly PCGF2/4) at its core, lacks the CBX and PHC subunits and instead associates with RYBP or YAF2 that stimulates RING1A/B catalytic activity and is thus responsible for the preponderance of in vivo H2AK119 ubiquitylation (Blackledge et al., 2014; Rose et al., 2016; Fursova et al., 2019). Depending on the PCGF protein, ncPRC1 also associates with additional accessory factors that fine-tune its catalytic activity and recruitment mechanism.

The canonical function of PRC2 in depositing H3K27me3 has remained undisputed since its discovery (Cao et al., 2002; Czermin et al., 2002; Kuzmichev et al., 2002), but its association with accessory factors classifies it into PRC2.1 and PRC2.2. PRC2.1 contains Elongin BC and Polycomb repressive complex 2-associated protein (EPOP) or PALI1/2 and one of the PCL proteins (PCL1/2/3) that directs its recruitment to unmethylated CpG islands (CGIs) (Li et al., 2017; Perino et al., 2018), whereas PRC2.2 contains JARID2 and AEBP2 that recruit it to ncPRC1-deposited H2AK119ub-containing nucleosomes and promote its methyltransferase activity (Kasinath et al., 2021). The diversity in composition and functional mechanisms of PRC1 and PRC2 have been extensively reviewed by Blackledge and Klose. (2021) and Kim and Kingston. (2022).

The discovery that PRC1, PRC2, and their deposited histone marks colocalize at developmentally regulated genes in *Drosophila* (Cao et al., 2002; Czermin et al., 2002; Tolhuis et al., 2006; Schwartz et al., 2006) and mammals (Boyer et al., 2006; Bracken et al., 2006) inaugurated a wealth of studies during the past two decades on the functional synergy between PRC1 and PRC2 in developmental contexts, which have yet to reach a complete consensus. More recently, the Polycomb repressive system has also been implicated in crosstalk with other epigenetic modifiers and their associated modifications. These discoveries pose numerous new directions in clinical and developmental biology research. While the colocalization of H3K27me3 and H3K4me3 at the bivalent promoters represents the best-known example of crosstalk (Macrae et al., 2023; Schuettengruber et al., 2017), in this review, we summarize the current knowledge about the less appreciated crosstalk mechanisms within and beyond the Polycomb repressive system, particularly focusing on Polycomb’s crosstalk with H3K9, H3K36, and DNA methylation, and we highlight major conceptual gaps that await clarification by future studies.

## Crosstalk within the Polycomb repressive system and with CGIs

**Figure 1.**
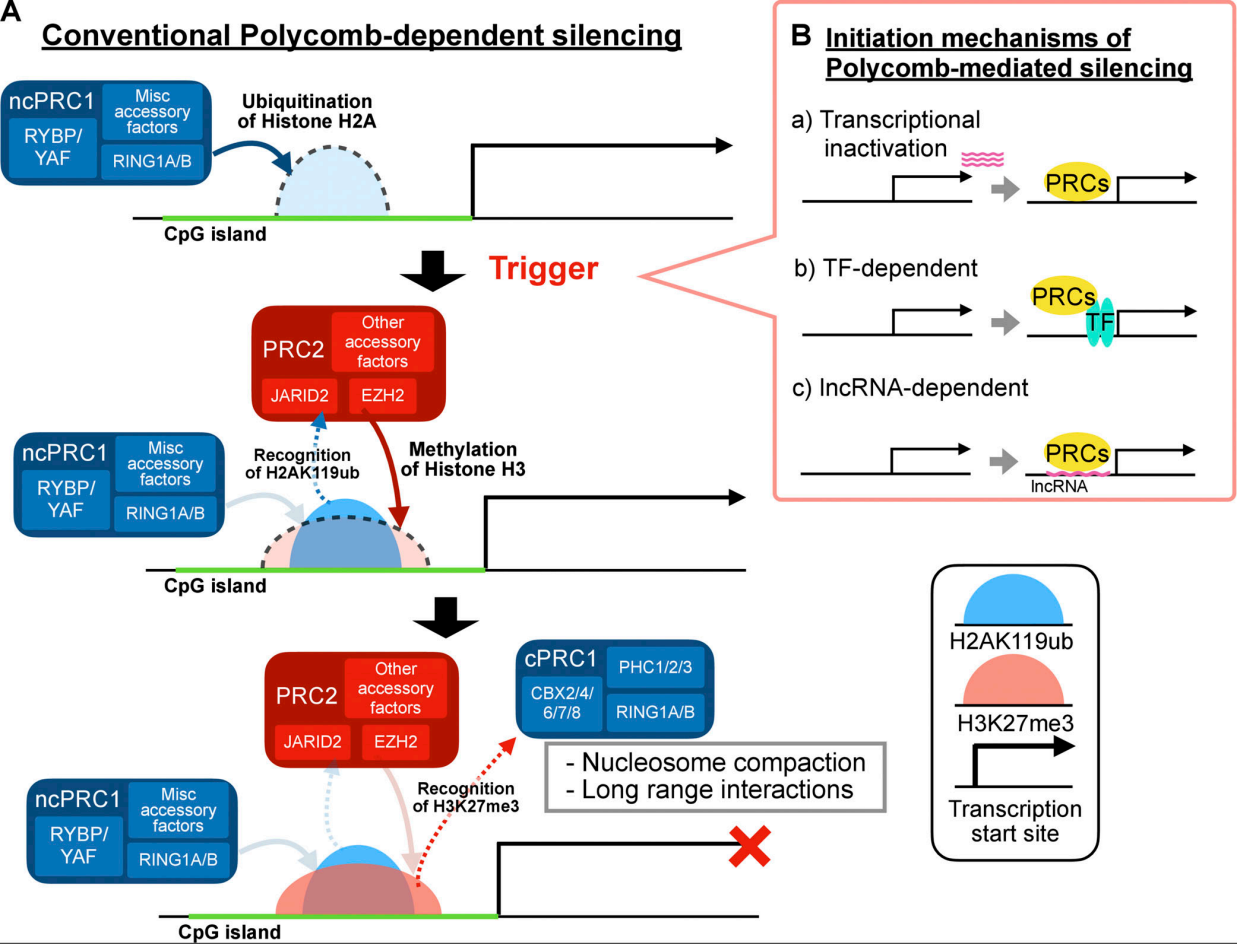
**The canonical mode of action of the Polycomb repressive system. (A)** ncPRC1 is recruited to target sites via mechanisms enabled by its miscellaneous accessory factors. The catalytic activity of RING1B is stimulated by the RYBP or YAF subunit, resulting in deposition of H2AK119ub. PRC2.1 and PRC2.2 are subsequently recruited, at least via the recognition of H2AK119ub by the JARID2 subunit of PRC2.2, and they deposit H3K27me3 via their catalytic subunit, EZH2. H3K27me3 in turn recruits cPRC1 via the chromodomain of cPRC1’s CBX subunit. cPRC1, which has very low in vivo ubiquitylation activity, promotes nucleosome compaction and long-range interactions between cPRC1-bound sites. The relative direct contribution of H2AK119ub, H3K37me3, and cPRC1-mediated chromatin interactions to gene repression appears to be context dependent and is not fully understood (Kim and Kingston, 2022). **(B)** Initiation mechanisms of PRCs binding to target genes. **(a)** Transcriptional inactivation promotes assembly of ncPRC1, especially ncPRC1.1, whose KDM2B subunit is already bound to CGI-associated promoters. H2AK119ub deposited by ncPRC1 then recruits PRC2. This is thought to be the most common mechanism of initiation of Polycomb-mediated repression of gene promoters in mammals. **(b)** PRCs can be recruited by transcription factors (TFs). For example, ncPRC1.6 is recruited by its subunit, the MGA/MAX heterodimer, to germline-specific genes that contain E-box motifs (Endoh et al., 2017). Pioneer TFs such as FOXA1/2/3 and OCT4 can cooperate with PRDM factors to recruit PRC1 to lineage-incompatible enhancers, likely by direct physical interaction with RING1B (Matsui et al., 2024). **(c)** ncPRC1.3/ncPRC1.5 can be recruited by chromosome-bound regulatory long non-coding RNA (lncRNA), for example, *XIST* on the inactivated X chromosome (Almeida et al., 2017; Pintacuda et al., 2017), and *AIRN* and *KCNQ1OT1* on autosomes (Schertzer et al., 2019). This interaction is mediated by the adaptor protein heterogeneous nuclear ribonucleoprotein K (HNRNPK) and results in formation of transcriptionally repressed domains at large chromosomal regions.

The discovery of H3K27me3-dependent recruitment of cPRC1 (Cao et al., 2002; Czermin et al., 2002; Kuzmichev et al., 2002) and the x-ray structure of Pc (the *Drosophila* ortholog of CBX) binding to H3K27me3 via its chromodomain (Min et al., 2003; Fischle et al., 2003) led to the initial hierarchical recruitment model in which PRC2 acts upstream of cPRC1. The subsequent discovery of ncPRC1 that lacks a CBX subunit (Wang et al., 2010; Gao et al., 2012), its H3K27me3-independent recruitment to Polycomb target sites (Tavares et al., 2012), and the ability of ectopically tethered ncPRC1-specific PCGF (PCGF1/3/5/6 but not PCGF2/4) to robustly recruit PRC2 and establish Polycomb domains de novo (Blackledge et al., 2014) collectively placed ncPRC1 upstream of PRC2 in the hierarchical recruitment model. This model is corroborated by additional studies, which identified the ubiquitin interaction motif (UIM) in the PRC2.2 accessory subunit JARID2 that binds to H2AK119ub (Cooper et al., 2016), illustrated the H2AK119ub-dependent recruitment and activation of PRC2.2 (Kalb et al., 2014) and its molecular basis (Kasinath et al., 2021), and demonstrated a paramount role for ncPRC1-mediated H2AK119ub in Polycomb domain establishment and gene repression in mouse embryonic stem cells (mESCs) (Endoh et al., 2012; Tamburri et al., 2020; Blackledge et al., 2020; Fursova et al., 2019; Dobrinić et al., 2021) and during ESC-to-EB (embryoid body) differentiation (Sugishita et al., 2021). In the physiological context, a critical role for H2AK119ub deposited by PCGF1- and PCGF6-containing ncPRC1 (ncPRC1.1/1.6) in guiding H3K27me3 deposition has been identified in the re-establishment of canonical, promoter-bound Polycomb domains following fertilization (Chen et al., 2021; Mei et al., 2021). In mESCs, H2AK119ub exhibits a much faster post-replication restoration kinetics than H3K27me3 (Flury et al., 2023), which is in line with the higher activity of JARID2-containing PRC2.2 in the G2 and of PRC2.1 in the G1 phase of the cell cycle (Asenjo et al., 2020). Taken together, there is compelling evidence that ncPRC1-mediated H2AK119ub deposition takes precedence in establishing and maintaining Polycomb domains, but there is still much room for new discoveries regarding upstream mechanisms that recruit ncPRC1 and downstream mechanisms that contribute to gene regulation (Fig. 1; Kim and Kingston, 2022).

PRCs can be recruited to germline-specific genes (Endoh et al., 2017) and lineage-incompatible enhancers (Matsui et al., 2024) by transcription factors, and can form large repressive domains by engagement with chromatin-bound long non-coding RNA, such as *XIST*, (Almeida et al., 2017; Pintacuda et al., 2017), *AIRN*, and *KCNQ1OT1* (Schertzer et al., 2019), which is mediated by heterogeneous nuclear ribonucleoprotein K (HNRNPK) (Fig. 1 B). However, CGIs and transcription inactivity represent the most typical strategy for targeting PRCs to gene promoters in mammals (Fig. 1). The majority of mammalian Polycomb targets reside in CGIs (Ku et al., 2008; Mikkelsen et al., 2007) and they appear to recruit PRC2 when being transcriptionally inactive (Riising et al., 2014). Ectopically inserted CGIs were also demonstrated to be sufficient for PRC2 recruitment (Jermann et al., 2014; Mendenhall et al., 2010; Wachter et al., 2014). Conversely, in oocytes, preimplantation embryos (Zheng et al., 2016), naïve ESCs (Marks et al., 2012; Joshi et al., 2015; McLaughlin et al., 2019), and *DNMT1* knockout (KO) mouse embryonic fibroblasts (Reddington et al., 2013), the global DNA hypomethylation results in redistribution of PRC2 away from its canonical CGI targets sites. Consistent with ncPRC1 being placed at the most upstream of the hierarchical recruitment model, ncPRC1.1 contains a KDM2B subunit that specifically recognizes unmethylated CpG through its CXXC domain (Farcas et al., 2012; Wu et al., 2013; He et al., 2013). KDM2B binds to almost all CGIs in mESCs regardless of their transcriptional status, and transcription inactivation triggers recruitment of other ncPRC1.1 components and thus assembly of functional ncPRC1.1 complexes to establish de novo Polycomb-mediated repression, at least during ESC-to-EB differentiation (Sugishita et al., 2021). PRC2.2 recruitment depends on the recognition of H2AK119ub by JARID2, whereas recruitment of PRC2.1 depends on its PCL subunit (Healy et al., 2019; Glancy et al., 2023; Li et al., 2017), whose winged-helix domain also specifically recognizes unmethylated CpG (Li et al., 2017). In mESCs, either *PCL1/2/3 *triple KO (tKO) or *JARID2* KO only partially reduces PRC2 and H3K27me3 levels at Polycomb target sites, with *JARID2* KO causing a milder effect than *PCL1/2/3* tKO, and their combined quadruple KO is required to abolish H3K27me3 deposition (Healy et al., 2019). This indicates that PRC2.1 and PRC2.2 are recruited in parallel, by PCL-mediated CGI recognition and JARID2-mediated H2AK119ub recognition, respectively. Interestingly, whereas *JARID2* KO resulted in the expected retention of SUZ12 peaks that align precisely with CGIs due to PCL-PRC2.1 activity (Glancy et al., 2023), SUZ12 peaks decayed rapidly and proportionally upon proteolytic depletion of PRC1 via an auxin-inducible degron (Dobrinić et al., 2021). This implies that, in addition to recognition of CGI by the PCL subunit, PRC2.1 recruitment also depends on H2AK119ub, a requirement that has not yet been defined.

It has remained undisputed that H3K27me3 recognition by the CBX subunit drives cPRC1 recruitment downstream of PRC2. However, finer control of this process has been demonstrated by a recent study that revealed distinct roles of PRC2.1 and PRC2.2 in the recruitment of cPRC1 (Glancy et al., 2023). The PRC2.2 subunit JARID2 is specifically required, in addition to H3K27me3, for the recruitment of CBX7-cPRC1, while other cPRC1 formations are more reliant on high levels of H3K27me3 deposited by PRC2.1 for their recruitment (Glancy et al., 2023). Although the exact mechanism of JARID2-dependent recruitment of CBX7 remains unclear, a coimmunoprecipitation followed by mass spectrometry experiment suggested a specific association of CBX7 but not CBX2 with JARID2 (Jaensch et al., 2021). Circular chromosome conformation capture sequencing at the *TBX3* promoter reveals that upon differentiation of mESCs to pregastrulation epiblast-like cells (EpiLCs), the *TBX3* promoter gained specific long-range contacts with three distant sites with prebound PRC2, which is accompanied by CBX7 gain at the three distant sites, gain of Polycomb occupancy at the *TBX3* promoter, and downregulation of *TBX3* expression. De novo CBX7-cPRC1 recruitment and long-range contacts specifically depends on JARID2-PRC2.2 but not PCL-PRC2.1. However, despite almost intact de novo long-range interactions at the *TBX3* locus, *PCL1/2/3* tKO resulted in more significant impairment of repression of *TBX3* and other PRC2 target genes newly acquired upon ESC-to-EpiLC differentiation (Glancy et al., 2023).

Despite cPRC1-mediated long-range interactions being detected in ESCs by Hi-C experiments (Schoenfelder et al., 2015; Kundu et al., 2017; Bonev et al., 2017; McLaughlin et al., 2019) and their dynamic changes during differentiation being demonstrated (Kundu et al., 2017; Bonev et al., 2017; McLaughlin et al., 2019), their role in gene regulation appears context-dependent. While some studies demonstrated the anticipated role in gene repression (Ogiyama et al., 2018; Ngan et al., 2020), others suggested that Polycomb long-range interactions could represent poised enhancer–promoter interactions that allow rapid induction of gene expression at later developmental stages (Kondo et al., 2014; Cruz-Molina et al., 2017; Pachano et al., 2021; Ngan et al., 2020; Loubiere et al., 2020). Yet another study showed that, while CKM–Mediator bridges long-range interactions between CBX7-cPRC1 bound sites in ESCs, it is the CKM–Mediator, not the cPRC1 long-range interactions, that contributes to gene activation during retinoic acid–induced differentiation (Dimitrova et al., 2022). Thus, the functional significance of JARID2-dependent CBX7 recruitment and long-range interaction formation requires further examination in additional biological contexts.

## Interactions with H3K4me3

H3K4 methylation (H3K4me1/2/3), a hallmark of active promoters and enhancers, is deposited by six methyltransferases of the KMT2 family. In particular, KMT2A/B and PRC2 share many target promoters, where they are generally considered to antagonize each other (Macrae et al., 2023). However, recent studies also demonstrated the roles of cofactors MEN1 and EPOP associated with KMT2A/B and PRC2.1, respectively, in enabling non-canonical means by which these two histone marks interact with each other.

**Figure 2.**
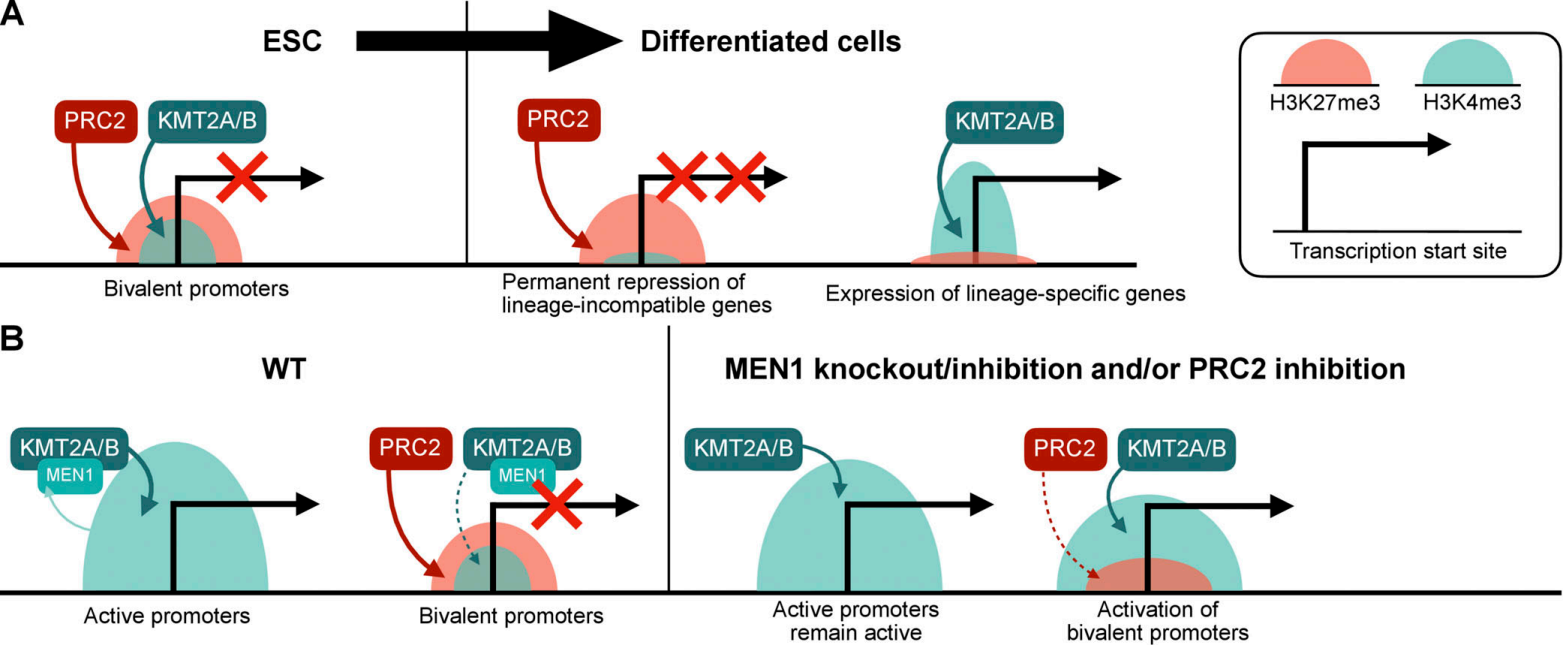
**Crosstalk with H3K4 methylation. (A)** Bivalent promoters comarked with H3K27me3 and H3K4me3 are abundant in ESCs, in which they are temporarily repressed but are later resolved into monovalent promoters possessing either the active H3K4me3 mark or the repressive H3k27me3 mark. Despite this general trend, differentiated cells also possess bivalent promoters, and this resolution is not always absolute; transcription activity depends on the relative levels of H3K4me3 of H3K27me3 (Jadhav et al., 2016, 2020). **(B)** While the H3K4me3 mark deposited by KMT2A/B is antagonistic to the H3K27me3 mark deposited by PRC2 at bivalent promoters, KMT2A/B is more robustly recruited to active monovalent promoters by its MEN1 accessory factor. MEN1 inhibition or KO thus augments KMT2A/B recruitment to bivalent promoters and promotes gene repression. PRC2 inhibition has the same effect on bivalent promoters, and the combined inhibition of PRC2 and MEN1 leads to more effective activation of bivalent promoters (Sparbier et al., 2023).

The canonical antagonism between H3K27me3 and H3K4me3 is manifested at bivalent promoters, where H3K27me3 and H3K4me3 colocalize. Bivalent promoters are thought to mainly act in early developmental stages to poise lineage-specific genes for activation at later developmental stages (Bernstein et al., 2006; Macrae et al., 2023). During development, the H3K27me3 dynamics at bivalent promoters inversely correlate with H3K4me3 so that H3K4me3 levels decrease over time at promoters that gain H3K27me3, and H3K4me3 levels increase at promoters that lose H3K27me3, as seen in maturing cerebellar granule neurons, for example (Ramesh et al., 2023). As development proceeds, some bivalent genes gradually resolve into monovalent genes marked with either H3K4me3 or H3K27me3, which correspond to lineage-specific and lineage-incompatible genes, respectively (Fig. 2 A). However, adult tissues still retain some bivalent, lineage-incompatible genes that require sustained Polycomb activity for proper repression (Jadhav et al., 2016, 2020). Bivalent promoters represent the best-known example of Polycomb crosstalk, and we refer readers to an excellent recent review dedicated to this subject (Macrae et al., 2023), which highlights a paucity of studies regarding the role of PRC1 in the context of bivalent promoters despite the fact that 40% of bivalent promoters are bound by PRC1 in ESCs. Thus, how ncPRC1 and cPRC1 occupancy changes at bivalent promoters and whether PRC1 mediates the function of bivalent promoters will be important topics to explore in future studies.

More recently, an unexpected synergy between MEN1, an accessory component of KMT2A/B, and Polycomb complexes PRC2.1 and PRC1.1, in repressing bivalently modified major histocompatibility complex I (MHC-I) genes and thus restricting their expression upon interferon-γ treatment was identified (Sparbier et al., 2023). MEN1 targets KMT2A/B to active, H3K4me3-monovalent loci and thus away from bivalent promoters, making the latter more accessible to PRC2.1 (Fig. 2 B). Combined inhibition of PRC2 and MEN1 reactivated MHC-I expression in small-cell lung cancer (SCLC) tumors and improved cytotoxic T cell–mediated killing in SCLC mouse models. PRC2-MEN1 synergy was independently identified in another co-dependency mapping experiment using pan-cancer cell lines. For diffuse large B cell lymphomas cells with *EZH2* gain-of-function mutations that are resistant to EZH2 inhibitor treatment alone, combined inhibition of EZH2 and MEN1 effectively inhibited their proliferation (Chen et al., 2023).

EPOP, initially discovered as an accessory factor of PRC2.1 that is mutually exclusive with PALI1 (Alekseyenko et al., 2014), was later identified as a generic CGI binding protein having a dual role in modulating H3K27me3 and H3K4me3 levels, as its knockdown increases H3K27me3 levels at PRC2-rich CGIs, but leads to a focusing of H3K4me3 at PRC2-poor CGIs (Liefke and Shi, 2015). In addition to PRC2, EPOP also independently interacts with the transcription elongation factor Elongin BC and the H2A deubiquitinase USP7, both of which are generally associated with active genes marked with broad H3K4me3 domains. While the majority of EPOP is associated with such active genes, *EPOP* KO has only minor impact on these genes and instead causes significant de-repression of PRC2 targets (Liefke et al., 2016). *EPOP* has been identified as a putative oncogene (Liefke et al., 2016), and systematic CRISPR screening experiments implicate it as one of the “common core essential” genes required for efficient proliferation of most types of cancer cells (Meyers et al., 2017). *EPOP* expression is high in ESCs and is generally downregulated during development, which might be due to the fact that the *EPOP* gene is a direct target of several pluripotency factors, including NANOG and SOX2 (Liefke et al., 2016). However, *EPOP* is also specifically highly expressed in the cerebral cortex in adults (Liefke and Shi, 2015; Lonsdale et al., 2013). The exact role of EPOP in development and carcinogenesis remains obscure, and it is unknown whether its property of concomitant regulation of H3K27me3 and H3K4me3 contributes to these processes.

## Interactions with H3K36 methylation

H3K36 di- and trimethylation (H3K36me2/3), which are mainly deposited by NSD1/2 and SETD2, respectively, are generally associated with euchromatin and active transcription (Wagner and Carpenter, 2012; Morris et al., 2005; Krogan et al., 2003). Whereas H3K36me3 is usually associated with promoters, H3K36me2 occupies gene bodies and broad intergenic regions. Like H3K4me3, they are also “active marks” and are thus generally antagonistic to the Polycomb repressive system. However, due to the different genomic distribution of H3K36me2 and H3K36me3 as well as the division of labor between the enzymes that dimethylate and trimethylate H3K36, the interactions of H3K36me2/3 with Polycomb are manifold.

**Figure 3.**
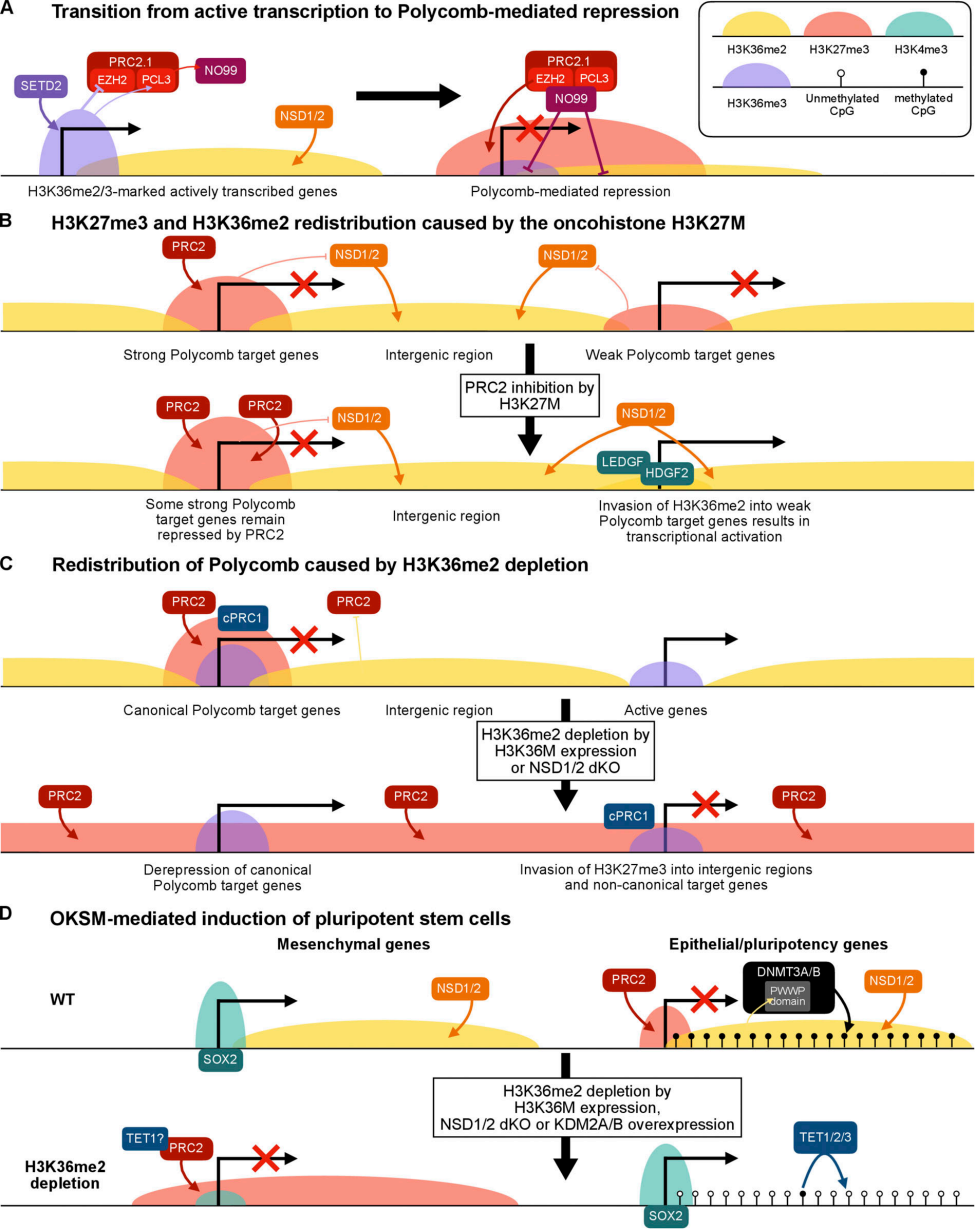
**Crosstalk with H3K36 methylation. (A)** Although PCL1/2/3 all possess a Tudor domain that recognizes H3K36me3, H3K36me2/3 inhibits EZH2 catalytic activity, and thus H3K27me3 and H3K36me2/3 usually do not colocalize. PCL3 is unique in that it was shown to be able to recruit NO66, a H3K36me2/3 demethylase, and thus could potentially help to initiate PRC2-mediated repression of hitherto H3K36me2/3 marked, actively transcribed genes. **(B)** The oncohistone H3K27M inhibits PRC2 activity and thus depletes H3K27me3 at weak PRC2 targets. Invasion of H3K36me2, which is deposited by NSD1/2, into these regions results in transcription activation, which is mediated at least in part by LEDGF and HDGF2. **(C)** *NSD1/2* double KO largely recapitulates the effect of H3K36M overexpression in that they both deplete intergenic H3K36me2, resulting in redistribution of PRC2 from their canonical targets to intergenic regions and non-canonical genic targets and hence their up- and downregulation, respectively (Rajagopalan et al., 2021). **(D)** H3K36me2 depletion improves iPSC induction efficiency by promoting the simultaneous silencing of mesenchymal genes and activation of epithelial/pluripotency genes. Epithelial/pluripotency gene activation depends on the DNA hypomethylation facilitated by reduced DNMT3A/B recruitment and increased TET activity. Loss of H3K36me2 promotes PRC2 recruitment and thus silencing of mesenchymal genes (Hoetker et al., 2023). Additionally, the catalytic-independent activity of TET1 (Chrysanthou et al., 2022) may also contribute to PRC2 recruitment.

The antagonism between H3K27me3 and H3K36me2/3 is a well-evidenced phenomenon as they do not colocalize on chromatin, and the presence of one mark inhibits deposition of its counterpart in vitro (Schmitges et al., 2011; Yuan et al., 2011; Zheng et al., 2012; Finogenova et al., 2020) and in vivo (Streubel et al., 2018; Chaouch et al., 2021; Lu et al., 2016). The PCL proteins of PRC2.1 each contain a Tudor domain that forms a hydrophobic cage with a high affinity for H3K36me3 (Musselman et al., 2012; Ballaré et al., 2012; Cai et al., 2013; Brien et al., 2012; Qin et al., 2013). This PCL-mediated recognition of H3K36me3 is thought to inhibit PRC2 catalytic activity, (Musselman et al., 2012; Finogenova et al., 2020), and unmethylated H3K36 is required for the proper positioning of H3K27 at the catalytic center of PRC2 (Finogenova et al., 2020). Conversely, PCL3 (PHF19) associates with NO66, a H3K36me2/3 demethylase (Sinha et al., 2010), pointing to the possibility that PCL3–PRC2.1 is recruited to some hitherto actively transcribed, H3K36me3-marked genes to initiate their repression by coordinated H3K36 demethylation and H3K27 trimethylation (Fig. 3 A; Brien et al., 2012). PCL3 is upregulated during ESC-to-neural progenitor cell differentiation (Kloet et al., 2016) and is the most frequently reported PCL protein to be implicated as an oncogene (Fischer and Liefke, 2023). Whether its unique role in recruiting NO66 has relevance in these developmental and pathological contexts, and the mechanism by which PCL3 selects its targets out of all H3K36me3-marked regions, requires further exploration.

Our understanding of the dynamic interplay between H3K27me3 and H3K36me2/3 has been advanced by recent studies on the H3 oncohistone bearing the K27M or K36M mutation (H3K27M/H3K36M), which acts in a dominant-negative fashion to attenuate but not completely abolish H3K27me3 or H3K36me2/3, respectively (Mitchener and Muir, 2022; Lewis et al., 2013; Lu et al., 2016). H3K27M is a key driver of diffuse intrinsic pontine gliomas (DIPGs) (Phillips et al., 2020). It acts by causing contraction of H3K27me3 domains and corresponding expansion of H3K36me2 domains (Stafford et al., 2018) due to NSD1 and NSD2 activity (Yu et al., 2021). The expanded H3K36me2 domains are subsequently recognized by LEDGF and HDGF2, which functionally resemble the FACT complex to facilitate RNA polymerase II–mediated transcription (LeRoy et al., 2019), resulting in activation of protumorigenic pathways, such as epidermal growth factor receptor and CHEK2 signaling (Yu et al. [2021]; Fig. 3 B). Loss of either NSD1/2 or LEDGF/HDGF2 in DIPG cells impedes cellular proliferation and tumorigenesis and extends survival by delaying tumor onset in a xenograft mouse model (Yu et al., 2021). *NSD1/2* double KO barely affected the proliferation of human embryonic kidney 293 (HEK293T) cells but rendered DIPG cells inviable, further emphasizing the specific dependence of DIPG on H3K36me2 (Yu et al., 2021). Impaired PRC2 function and global reduction of H3K27me3 levels were also found in other tumors, especially those of the nervous system. For example, loss-of-function of PRC2 core subunits frequently occurs in peripheral nerve sheath tumors (Lee et al., 2014), and EZHIP (EZH inhibitory protein) is overexpressed in posterior fossa type A ependymoma (Pajtler et al., 2018). Notably, EZHIP inhibits PRC2 activity in a similar manner to H3K27M (Hübner et al., 2019; Jain et al., 2019; Piunti et al., 2019). It will be worth exploring the potential protumorigenic roles of H3K36me2 that are expected to be upregulated in these PRC2-deficient contexts other than H3K27M.

Similar to H3K27M, H3K36M has a dominant negative effect to partially inhibit H3K36me2/3, and has been reported to impair ES-to-EB differentiation, hematopoiesis, adipogenesis, myogenesis, chondrogenesis, and osteogenesis (Brumbaugh et al., 2019; Zhuang et al., 2018; Lu et al., 2016). Consistent with H3K36me2/3 antagonizing PRC2, in H3K36M-expressing or *NSD2* KO preadipocytes, which fail to differentiate into adipocytes, many genes are associated with decreased H3K36me2, elevated H3K27me3, and decreased expression compared with successfully differentiated adipocytes. These include the master adipogenic transcription factor C/EBP α, whose inhibition explains the differentiation defects (Zhuang et al., 2018). Likewise, a subset of H3K27me3/H3K36me3 co-marked genes that lose H3K36me3 upon H3K36M expression in hematopoietic stem and progenitor cell show increased H3K27me3 associated with the promoter and gene body, which include several key hematopoietic regulators that became downregulated. However, H3K36M does not completely deplete H3K36me3, and at those H3K27me3/H3K36me3 comarked genes whose H3K36me3 remain unchanged, H3K27me3 is attenuated and expression is upregulated (Brumbaugh et al., 2019), reflecting the relocalization of limited amount of PRC2 from canonical targets to ectopic targets that become amenable to PRC2 activity following attenuation of H3K36me2/3.

The effects of H3K36M on PRC2 redistribution and transcriptomic changes largely parallel with *NSD1/2* deletion in mesenchymal stem cells (MSCs), wherein SETD2 maintained H3K36me3 at loci comarked with H3K27me3, but PRC2 were redirected to H3K36me2/H3K27me3 comarked loci that lost H3K36me2 due to *NSD1/2* KO (Rajagopalan et al., 2021). cPRC1 redistribution ensues H3K27me3 redistribution, resulting in derepression of canonical Polycomb targets (Lu et al., 2016; Rajagopalan et al., 2021), which include MSC genes that promote multipotency and sarcomagenesis and thus impair normal osteogenesis and adipogenesis (Lu et al., 2016). The PRC2 redistribution and differentiation defects exhibited by mesenchymal cells expressing H3K36M were largely recapitulated by *NSD1/2* KO but not *SETD2* KO (Rajagopalan et al., 2021; Zhuang et al., 2018). Conversely, the overexpressed NSD2 in multiple myeloma causes global gain of H3K36me2 and reduction of H3K27me3. However, at some individual loci, more focused PRC2 activity leads to increased H3K27me3 and thus gene repression (Popovic et al., 2014). These findings suggest that the PRC2 redistribution caused by perturbation of H3K36 methylation is mainly dependent on H3K36me2, not H3K36me3, which is further supported by the notion that NSD1, but not SETD2, is specifically required to safeguard a subset of genes crucial for spermatogenesis against H3K27me3-mediated repression (Shirane et al. (2020); Fig. 3 C). In some contexts, however, *SETD2* KO also impairs hematopoiesis (Zhang et al., 2018), and combined *NSD1/NSD2/SETD2* tKO was required to impair chondrocyte differentiation of MSCs (Lu et al., 2016).

Taken together, these studies demonstrate that, upon H3K36me2/3 depletion, PRC2 is redirected from canonical to ectopic targets, resulting in perturbation of gene expression in both directions that eventually leads to differentiation defects or contributes to oncogenesis. The loss of H3K36me3 located at promoters and H3K36me2 located at broad intergenic and genic regions both play a role in this process, but their relative contribution is context dependent. Interestingly, most of the observed bidirectional changes in the H3K27me3 landscape came from studies on cells of the mesodermal lineage. In mESCs, however, H3K36me3 depletion in mESCs resulted in unidirectional global elevation of H3K27me3 levels including at H3K27me3 premarked loci (Streubel et al., 2018; Chen et al., 2022). A possible cause for the lack of redistribution effect might be the exceptionally high abundance of PRC2 (Kloet et al., 2016), reinforced by repression of its endogenous antagonist, EZHIP, in mESCs (Piunti et al., 2019; Albert et al., 2023, *Preprint*), but this requires further verification.

The ability of H3K36M expression and H3K36me2 depletion to promote stemness and inhibit mesenchymal differentiation via redistribution of PcG proteins observed by Lu et al. (2016) was further corroborated by the extraordinary ability of H3K36M to promote OKSM (OCT4, KLF4, SOX2, MYC)-mediated induced pluripotent stem cell (iPSC) formation from mouse embryonic fibroblasts (MEFs) by inactivating mesenchymal gene expression while simultaneously promoting epithelial/pluripotency gene expression, an effect that was largely recapitulated by overexpression of KDM2A, a H3K36me2-specific demethylase (Hoetker et al., 2023). As expected, increased H3K27me3 deposition by PRC2 mediates the repression of TGF β-responsive mesenchymal genes. In contrast, activation of epithelial/pluripotency gene was shown to be dependent on SOX2 binding to pluripotency-specific enhancers that are normally CpG-hypermethylated in MEFs but become CpG-hypomethylated upon H3K36M expression in a manner that depends on ten-eleven translocation (TET) proteins, which are methylcytosine dioxygenases involved in DNA demethylation (Hoetker et al., 2023).

H3K36me2/3 recruits the de novo DNA methyltransferases DNMT3A/B to intergenic and genic regions, respectively (Baubec et al., 2015; Weinberg et al., 2019; Yano et al., 2022; Chen et al., 2022), and thus H3K36me2/3 depletion attenuates DNA methylation and facilitates TET-dependent demethylation and activation of pluripotency genes (Hoetker et al., 2023). By inhibiting TET activity using dimethyloxalylglycine, Hoetker et al. (2023) showed that TET was involved in upregulation of epithelial/pluripotency genes but not downregulation of mesenchymal genes during iPSC induction of H3K36M-expressing MEFs (Fig. 3 D). However, an interesting alternative scenario could be that the catalytic-independent activity of TET1 represses these genes by recruiting PRC2 and the H3K27ac-specific deacetylase SIN3A (Chrysanthou et al., 2022; Wu et al., 2011; Williams et al., 2011; Neri et al., 2013; Li et al., 2018). Consistent with TET1 having an anti-mesenchymal role, catalytically dead TET1 was sufficient to recruit PRC2 and SIN3A to H3K4me3/H3K27me3 bivalent promoters in ESCs and restrict differentiation toward mesendoderm and trophectoderm (Chrysanthou et al., 2022). Clearly, the dynamic interplay between H3K36me2/3, H3K27me3, and DNA methylation in additional developmental and pathophysiological contexts beyond reprogramming and differentiation of ESCs and mesenchymal cells deserves further exploration.

## Crosstalk with DNA methylation and H3K9 methylation

Apart from the H3K27me3 and H2AK119ub marks deposited by Polycomb repressive complexes, DNA methylation at CpG motifs (DNAme) and H3K9 di- and tri-methylation (H3K9me2/3) are also representative repressive epigenetic marks (Padeken et al., 2022; Greenberg and Bourc’his, 2019). However, as PcG proteins are typically recruited to gene promoters located within unmethylated CGIs, they usually do not colocalize with DNAme or H3K9me2/3, as the latter marks are typically involved in the establishment and maintenance of the gene-poor constitutive heterochromatin. Despite this, at a small subset of genes in certain developmental contexts, Polycomb has been implicated in crosstalk with DNAme and H3K9me3.

**Figure 4.**
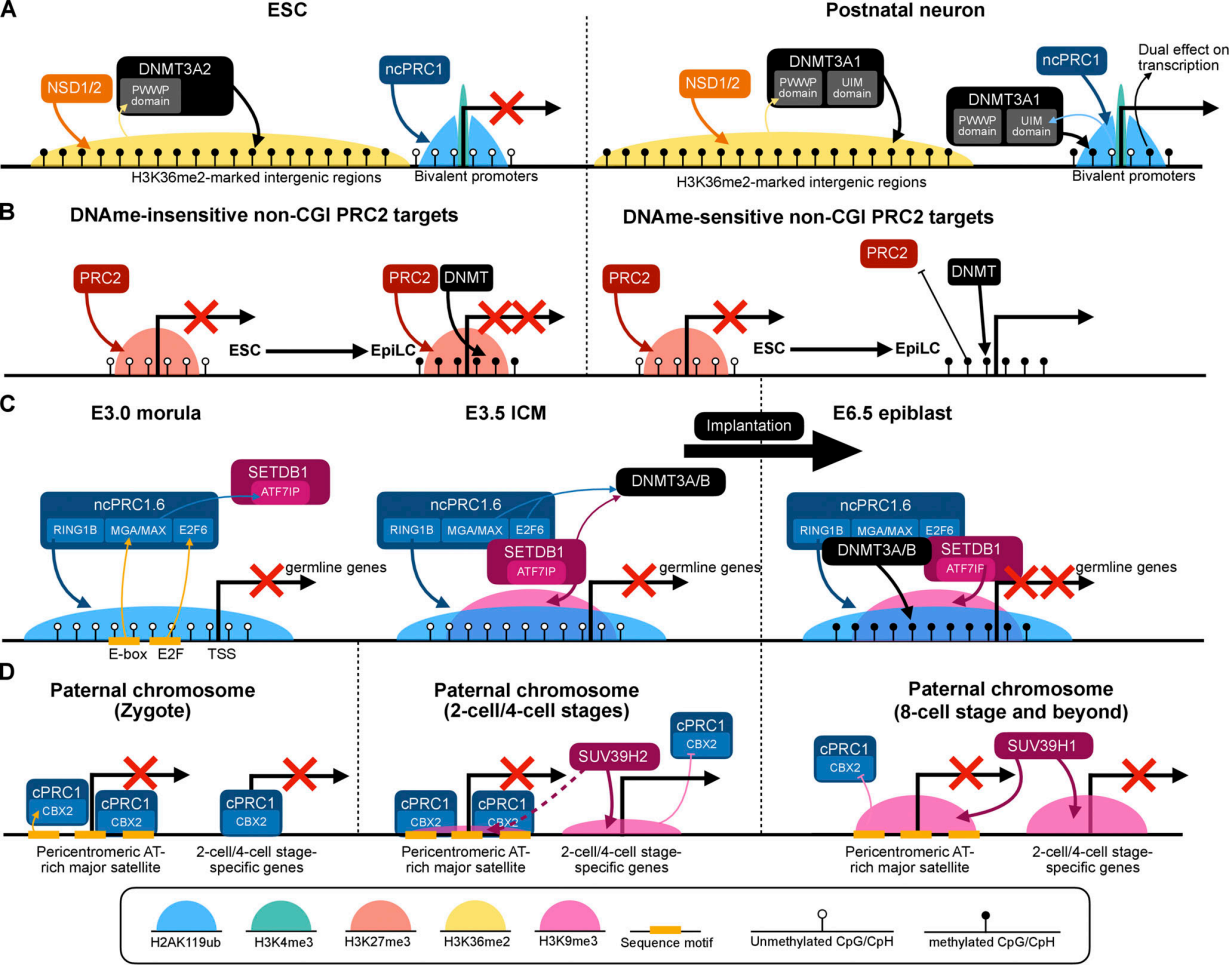
**Crosstalk with DNAme and H3K9me3. (A)** The short isoform of DNMT3A (DNMT3A2) is expressed in ESCs and it is targeted to H3K36me2-marked intergenic regions via its PWWP domain. The long isoform (DNMT3A1), which is the predominant form in specific adult cell types, including neurons but not glia, additionally processes a UIM domain that targets it to H2AK119ub-marked flanking regions of bivalent promoters. Loss-of-function of this domain results in hypomethylation of these promoters concomitant with both up- and downregulation of gene expression (Gu et al., 2022). **(B)** During ESC-to-EpiLC differentiation, some non-CGI PRC2 targets acquire de novo DNAme. DNAme evicts PRC2 at some sites (right) but not at the others (left), providing an explanation for the dual role of DNAme in transcription regulation (Albert et al., 2023, *Preprint*). **(C)** Germline genes are repressed by default and are only activated during germ cell development. Before implantation, this repression depends on ncPRC1.6 and SETDB1. ncPRC1.6 is recruited by sequence-specific recognition of E-box and E2F motifs by its MGA/MAX and E2F6 subunits, respectively, and SETDB1 recruitment at least partially depends on ncPRC1.6. ncPRC1.6 and SETDB1 are required for efficient recruitment of DNMT3A/B, which establishes post-implantation de novo DNA methylation that contributes to long-term repression of germ-line genes (Mochizuki et al., 2021). **(D)** While the maternal PCH is consistently marked by H3K9me3 that represses the transcription of the underlying major satellite sequences, the patPCH is initially devoid of H3K9me3 and instead repressed by cPRC1 upon fertilization. The gradual accumulation of H3K9me3 deposited initially by SUV39H2 and then by SUV39H1 at patPCH evicts cPRC1, resulting in a transition from cPRC1-dependent to H3K9me3-dependent repression of pericentromeric major satellites. However, the initial H3K9me3 deposited by SUV39H2 appears to be compatible with gene expression and might be required for the transient activation of some two-cell or four-cell stage-specific genes (Burton et al., 2020).

Polycomb-DNAme crosstalk has been implicated in the de novo DNA methylation of CGI promoters in the context of neuronal differentiation (Mohn et al., 2008), H3K27me3-mediated non-canonical genomic imprinting (Chen et al., 2019), microcephalic dwarfism (Heyn et al., 2019), and oncogenesis (Widschwendter et al., 2007; Schlesinger et al., 2007; Weinberg et al., 2021). Gene repression induced by ectopically tethered RYBP was maintained longer than was H2AK119ub after release of RYBP, with the repressed gene gaining DNAme, suggesting the possibility of ncPRC1/H2AK119ub-DNAme crosstalk (Zhao et al., 2020). Subsequently, this notion was validated and it involves recognition of H2AK119ub by a de novo DNA methyltransferase, DNMT3A1 (Fig. 4 A; Weinberg et al., 2021; Gu et al., 2022). When the H3K36me2/3-binding PWWP domain was mutated, DNMT3A1 accumulated at CGIs in a H2AK119ub dependent manner in MSCs (Weinberg et al., 2021), and targeting to H2AK119ub depends on the N-terminal UIM present in the long (DNMT3A1) but not the short (DNMT3A2) isoform of DNMT3A (Weinberg et al., 2021; Gu et al., 2022). DNMT3A1 is the predominant isoform in some postnatal cell types, including neurons but not glia, and moderate levels of recruitment of wild-type DNMT3A1 to flanking regions of bivalent promoters (Manzo et al., 2017; Gu et al., 2022) play important roles in postnatal development and are especially crucial for perinatal neurogenesis, presumably via activating and repressing different sets of genes (Gu et al., 2022; Wu et al., 2010). The regulation of H2AK119ub-dependent DNMT3A1 localization and the roles of its ramifications on DNAme landscape in more physiological and pathological contexts, such as the cytokine-induced de novo CGI DNAme (Spencer et al., 2017) and DNAme of Polycomb-regulated promoters during neuronal differentiation (Mohn et al., 2008), are worth further exploration.

Consistent with the notion that some hypomethylated genes upon DNMT KO are downregulated (Gu et al., 2022; Wu et al., 2010), the unorthodox role of DNAme in gene activation has been reported, and this role appears to be mediated by the well-established antagonism between Polycomb and DNAme. DNMT3A-mediated de novo DNAme evicts PRC2 from the non-promoter regulatory regions of some neural genes during neurogenesis and thus promotes their transcription (Wu et al., 2010; Ziller et al., 2018), and *FOXA2* appears to be regulated in a similar fashion during endoderm development (Bahar Halpern et al., 2014). During ES-to-EpiLC differentiation, the same mechanism is also responsible for the activation of a subset of H3K27me3 premarked genes, including *ZDBF2* and *CELSR2* (Fig. 4 B; Albert et al., 2023, *Preprint*). In the case of *ZDBF2* at least, the DNAme-dependent activation has life-long consequences (Greenberg et al., 2017). Targeted DNAme editing strategy based on catalytically inactive dCas9 fused to DNMT3A or TET1 further confirmed the direct role of de novo DNAme in PRC2 eviction and gene activation at specific loci (Ziller et al., 2018; Albert et al., 2023, *Preprint*). Interestingly, compared with CGIs, these sites usually have a lower CpG density (Albert et al., 2023, *Preprint*; Greenberg et al., 2017). Whether the Polycomb-DNAme switching represents a general gene regulatory mechanism at these non-CGI regions in a broader biological context requires further validation.

Despite CGIs being protected from DNAme during the wave of de novo DNA methylation concomitant with implantation, a small number of CGIs mainly associated with promoters of germline genes acquire DNA methylation after implantation, which is responsible for their long-term expression in somatic cells (Auclair et al., 2014). At the preimplantation stage, as well as in naïve ESCs (nESCs), these germline genes are also repressed despite being hypomethylated, and this repression depends on ncPRC1.6 and a H3K9me3-specific histone methyltransferase, SETDB1. Notably, both in vivo and in the in vitro nESC-to-EpiLC differentiation model, de novo DNA methylation depends on this initial ncPRC1.6 and SETDB1 occupancy, whereas PRC2 is dispensible (Fig. 4 C; Mochizuki et al., 2021; Al Adhami et al., 2023; Dahlet et al., 2021). Despite previous reports suggesting DNMT3B being responsible for de novo DNA methylation at germline genes and that the ncPRC1.6 component E2F6 being responsible for its recruitment (Velasco et al., 2010; Dahlet et al., 2021), another study demonstrated redundant roles for DNMT3A and DNMT3B (Auclair et al., 2014). In either case, the involvement of DNMT3B, which lacks a UIM, argues against the dependence on H2AK119ub for DNMT recruitment. In addition to E2F6, the ncPRC1.6 components MGA and MAX are also suggested to promote DNMT recruitment. Despite its negligible effect on H2AK119ub levels, ablation of the helix-loop-helix DNA-binding domain of MGA (*MGA*-ΔHLH) significantly abolishes de novo DNAme, and this effect was stronger than *PCGF6* KO (Mochizuki et al., 2021). Together with the previously reported coimmunoprecipitation of DNMT1, DNMT3A, DNMT3B, and DNMT3L with MAX in ESCs (Tatsumi et al., 2018), these findings point to a central role for the MGA/MAX heterodimer in recruiting DNMTs to ncPRC1.6 target sites, but whether this extends beyond germline genes requires further investigation.

As mentioned above, the initial DNAme-independent repression of germline genes relies on SETDB1 and ncPRC1.6, which show coenrichment along with their deposited H3K9me3 and H2AK119ub at these genes (Fig. 4 C; Mochizuki et al., 2021). H2AK119ub deposition temporally precedes H3K9me3 both in vivo and in the in vitro nESC-to-EpiLC differentiation model, and the non-canonical, KAP1/TRIM28-independent SETDB1 recruitment in nESCs and EpiLC to these germline genes depends on ncPRC1.6, as H3K9me3 levels are reduced by either *PCGF6* KO or *MGA*-ΔHLH. Because *MGA*-ΔHLH barely affects H2AK119ub levels, this recruitment depends on either MGA or the intact MGA-containing ncPRC1.6 complex but not H2AK119ub (Mochizuki et al., 2021). Supporting this notion, coimmunoprecipitation revealed interaction between PCGF6 and SETDB1 (Yang et al., 2016), between MAX and SETDB1 (Tatsumi et al., 2018), and between MAX and ATF7IP (a known binding partner of SETDB1) (Tsusaka et al., 2020) in ESCs. However, the exact domain on SETDB1 responsible for its interaction with ncPRC1.6 is yet to be defined, and whether this SETDB1/ncPRC1.6 synergy has functional significance beyond repression of germline genes is yet to be explored.

The transition from PRC1- to H3K9me3-mediated repression was also observed during early development at the paternal pericentromeric heterochromatin (patPCH), which, unlike its maternal counterpart, is devoid of H3K9me3 but instead bound by PRC1 at fertilization (Puschendorf et al., 2008; Tardat et al., 2015). The targeting of PRC1 depends on the AT-hook domain of CBX2 that recognizes the repetitive AT-rich major satellite sequences that constitute the PCH (Puschendorf et al., 2008; Tardat et al., 2015). PRC1 is subsequently replaced by H3K9me3, which accumulates at patPCH due to the activity of SUV39H2 (soon after fertilization) followed by SUV39H1 (after the eight-cell stage) (Burton et al., 2020). The exclusion of PRC1 is caused by heterochromatin protein 1 β bound to H3K9me3, which prevents the chromodomain of CBX from binding (Tardat et al., 2015). Immunofluorescence images showed that the de novo H3K9me3 on paternal chromosomes initially appears at chromosome arms before concentrating at the patPCH (Puschendorf et al., 2008; Burton et al., 2020). Also, it appears that the H3K9me3 deposited by SUV39H1 since the eight-cell stage, but not the initial H3K9me3 deposited by SUV39H2, represses transcription; instead, the initial SUV39H2 activity promotes the transient transcription of some genes during early development, especially at the two-cell stage (Burton et al., 2020). An interesting speculation is that the initial SUV39H2-deposited H3K9me3 acts to exclude PRC1 to achieve its gene-activating role. Thus, it would be worth to explore whether the genes downregulated at the two-cell stage upon SUV39H2 knockdown and those upregulated at the four-cell stage upon SUV39H1 overexpression (Burton et al., 2020) are repressed by cPRC1.

The mammalian H3K9me2-specific histone methyltransferase, G9A, has also been implicated in crosstalk with PRC2.1 (Alekseyenko et al., 2014; Conway et al., 2018). This is mediated by the PALI1 protein, which possesses separate PRC2 and G9A interacting domains (Conway et al., 2018). In prostate cancer cells with overexpressed PALI1, the G9A-PALI1-PRC2.1 supercomplex is formed, which mediates H3K9me2/H3K27me3 dual methylation at a set of promoters that are originally G9A targets, leading to gene repression that is more robust than could be caused by H3K9me2 alone (Fong et al., 2022). Consistent with this, in in vitro and xenograft models, inhibition of either PRC2 or G9A suppressed tumor growth, and their combined inhibition achieved even better efficacy (Fong et al., 2022).

## Conclusion and future perspectives

In the past few decades, much has been learned about epigenetic modifications by disturbing the enzymes responsible for their deposition (Millán-Zambrano et al., 2022; Greenberg and Bourc’his, 2019), and such studies established a critical role for the Polycomb system in the repression of developmentally regulated genes (Kim and Kingston, 2022). Meanwhile, as we have summarized in this review, it is becoming increasingly clear that Polycomb is involved in crosstalk with other epigenetic modifications, which has functional implications in development and diseases.

Much of the insight into the Polycomb crosstalk mechanisms originated from studies on cancers, a hallmark of which is drastically dysregulated epigenetic landscape that often involves a combination of multiple aberrant epigenetic modifications (Furth and Shema, 2022; Walton et al., 2023). Previous identification of oncogenic mutations of epigenetic modifiers has motivated the development of their corresponding small molecule inhibitors aimed to treat cancer, which include Tazemetostat, a PRC2 inhibitor recently approved by the FDA (Bates, 2020). While many of these drugs show effectiveness when applied alone, the growing body of knowledge regarding crosstalk mechanisms provides a conceptual framework for combinatorial therapy. Indeed, combined inhibition of PRC2 with G9A (Fong et al., 2022) or MEN1 (Sparbier et al., 2023; Chen et al., 2023) to antagonize cancers with PRC2 gain-of-function, and inhibition of the H3K36me2 pathway to antagonize cancers with PRC2 loss-of-function (Yu et al., 2021), both proved effective in in vitro and xenograft models. Additionally, synthetic lethality might be achieved, as exemplified by the hypersensitivity to DNA-hypomethylating agent in cancer cells with inactivated NSD1/2 or expression of the oncohistone H3.3K36M (Rajagopalan et al., 2021). Thus, further elucidation of the crosstalk mechanisms is of paramount medical relevance, as this knowledge improves our ability to decipher the precise etiology of the epigenomic aberrations observed in cancer cells and design effective therapeutic strategies to rectify them by leveraging the full potential of the growing repertoire of epigenome-targeting drugs.
